# A New Bivalve Species *Glauconome huangheensis* of the Genus *Glauconome* J. E. Gray, 1828 (Bivalvia, Venerida, Cyrenoidea, Glauconomidae), from Shandong, China

**DOI:** 10.3390/genes15121563

**Published:** 2024-12-01

**Authors:** Lingtong Kong, Yuxi Zhang, Cui Li, Zeyu Tang, Haiyan Wang

**Affiliations:** 1 College of Life Sciences, Qingdao Agricultural University, Qingdao 266109, China; kltong109@163.com (L.K.); zhang-yuxi@163.com (Y.Z.); 2 Department of Marine Organism Taxonomy & Phylogeny, Institute of Oceanology, Chinese Academy of Sciences, Qingdao 266071, China; licui@qdio.ac.cn (C.L.); tangzy@qdio.ac.cn (Z.T.); 3 University of Chinese Academy of Sciences, Beijing 100049, China

**Keywords:** Glauconomidae, *Glauconome*, *Glauconome angulata*, *Glauconome huangheensis* sp. nov., taxonomy, morphology

## Abstract

The family Glauconomidae has few species, limited molecular data description, and insufficient research attention. The biodiversity of Glauconomidae within China deserves further exploration. In recent years, the taxonomic status of Glauconomidae has undergone changes, and some studies have found a close relationship between Glauconomidae and the family Cyrenidae based on molecular data, suggesting that Glauconomidae should be classified under the superfamily Cyrenoidea. However, both domestic and international research has mainly focused on only four species of Glauconomidae, indicating an urgent need for more species data support. Recently, 46 specimens of Glauconomidae were collected in the Yellow River Estuary in Dongying City of Shandong Province in China. Through a comparative analysis of shell morphology and molecular phylogenetic analysis of *COI* and *16S rRNA*, two species of Glauconomidae was discovered. One is *Glauconome angulata* Reeve, 1844, and the other is a new species of Glauconomidae found in the Yellow River, named *Glauconome huangheensis* sp. nov. The *G. huangheensis* sp. nov. exhibits distinct differences in shell shape and shell color compared to other species of Glauconomidae, resembling *G. angulata*. There are also significant differences in shell color, shell sculpture, ligament size, and shell thickness. Furthermore, the molecular phylogenetic analysis based on *COI* and *16S rRNA* genes supports the validity of *G. huangheensis* sp. nov. as a species. It indicates a close phylogenetic relationship with *G. angulata*, making them sister species. This study provides a redescription of the morphological characteristics of *G. angulata* and *G. huangheensis* sp. nov., laying the foundation for the morphological classification, biodiversity research, and conservation of Glauconomidae species.

## 1. Introduction

Glauconomidae J. E. Gray, 1853, belongs to the phyla Mollusca, Bivalvia, Venerida, and Cyrenoidea. As of November 2024, there is 1 genus *Glauconome* J. E. Gray, 1828, and 12 species, 5 of which have partial molecular data records, including *G. angulata* Reeve, 1844, *Glauconome chinensis* J. E. Gray, 1828, *Glauconome rugosa* Hanley, 1843, *Glauconome straminea* Reeve, 1844, and *Glauconome virens* Linnaeus, 1767 [1].

Due to the difficulty in collecting samples and insufficient attention, there is limited research on the family Glauconomidae by scholars both domestically and internationally. Most of the existing studies are morphological classification articles based on morphological features and identification from several years ago. Molecular classification articles mainly focus on the systematic evolution of the Bivalvia, Venerida, or Veneridea, which includes 1–3 species of Glauconomidae, and mainly concentrate on 3 species, such as *G. chinensis*, *G. angulata*, and *G. rugosa* [2,3]. *G. chinensis* is mostly distributed in the middle and lower reaches of the Yangtze River and the sea area of Shantou, Guangdong, also known as “datou cheng”. *G. angulata* is mainly distributed in the waters of Liaoning, Hebei, and Shandong. *G. rugosa* has been collected and discovered in Vietnam, but no relevant reports have been found domestically [2,4]. There are some classification confusions and controversies regarding the family Glauconomidae in domestic research, and whether there are cryptic or new species in China remains to be studied [5,6]. Bieler’s analysis of the systematic evolution of the class Bivalvia found a closer relationship between the family Glauconomidae and the family Cyrenidae, so it was classified under the superfamily Cyrenoidea, using two species, *G. chinensis* and *G. rugosa*. However, there was an identification error in the use of *G. chinensis* based on the sample sequence collected from Japan in 2006 [2,3,5,6]. The validity of the family Glauconomidae’s classification is controversial, and it is urgent to discover cryptic species and increase the species coverage of the family Glauconomidae, combined with morphological and molecular data for verification.

In the “Catalogue of Marine Life in China” compiled by Academician Ruiyu Liu of the Institute of Oceanology, Chinese Academy of Sciences, in 2008, the family Glauconomidae was classified under the superfamily Glauconoidea of the order Venerida, including five species of *Glauconome* from China: *G. chinensis*, *Glauconome curta*, *Glauconome cerea*, *G. angulata* and *Glauconome primeana* (renamed as *G. angulata*), as well as one unidentified species, with only a brief introduction to their distribution and no morphological description [7]. In the “Illustrated Catalogue of Chinese Marine Bivalves”, published in 2008, Xu classified the family Glauconomidae under the superfamily Glauconoidea of Venerida based on morphological characteristics, including the same five species of *Glauconome* from China, and one unidentified species, identifying and introducing the species’ distribution and morphological description based on the external shell characteristics [8]. Some results, such as the identification of *G. curta* Reeve, 1844, are controversial, while the identification of *G. angulata* is consistent with this study. In the “Illustrated Guide to Marine Mollusks of China”, published in 2008, Zhang directly classified the family Glauconomidae under Venerida, without determining the superfamily level, including two species of *Glauconome* from China, among which the identification of *G. angulata* is controversial [9]. In “Species Resource Survey and Research in Haizhou Bay and Laizhou Bay,” Wang introduced one species of *Glauconome*, *G. primeana* (renamed as *G. angulata*), distributed in Haizhou Bay in Jiangsu Province, the Yellow Sea, and the Bohai Sea, living in the intertidal zone with freshwater inflow and sandy or muddy sediments [10]. In the “Illustrated Atlas of Common Marine Organisms in the Yellow River Delta Region” published by Hanzhen Zhang in 2018, one species of *Glauconome*, *G. primeana* (renamed as *G. angulata*), is included, obtained from the adjacent waters of the Yellow River Delta region, with the identification of *G. angulata* consistent with this study [11]. The validity of *G. angulata* at the Yellow River estuary can be confirmed, and, during morphological and molecular identification, samples of *Glauconome* with significant morphological differences were collected, with clear differences in *COI* and *16S* sequence data from *G. angulata*. After the analysis and validation of the validity of the new species, it was determined to be a new species of *Glauconome* in the family Glauconomidae, named *G. huangheensis* sp. nov., to recognize its discovery in the Yellow River estuary.

## 2. Material and Methods

**Figure 1:**
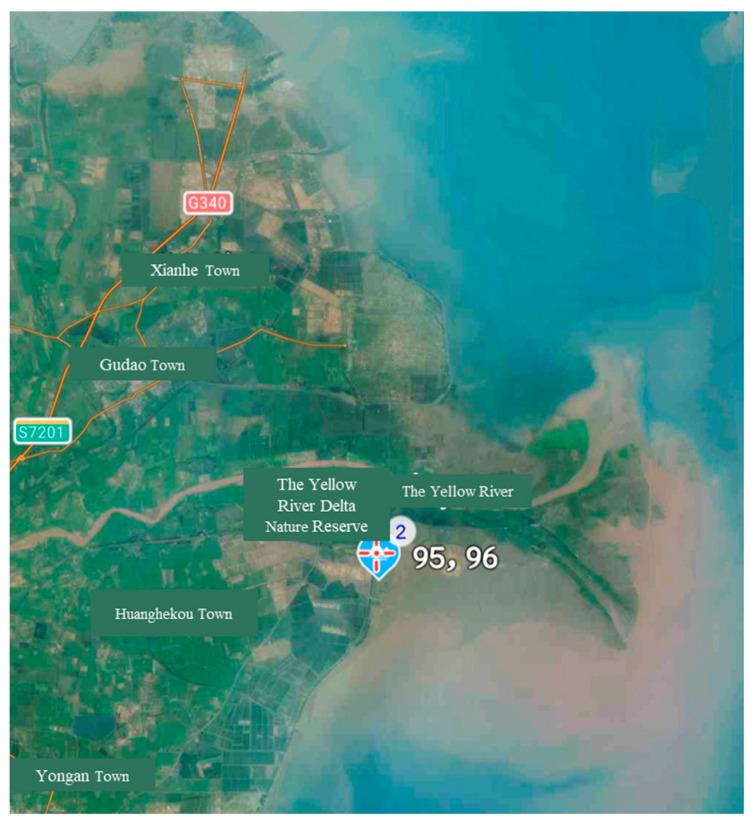
*G. huangheensis* sp. nov. sampling site coordinate map. The following can be observed: 95 site: 37.1704486° N, 119.02974214° E; 96 site: 37.1704486° N, 119.02974214° E.

The specimens were collected at sampling sites 95–96 in the Yellow River Delta Nature Reserve, Dongying City, Shandong Province, China, in May 2023 [Figure 1]. They were mainly collected from the intertidal zone of the Yellow River estuary. The specimens were photographed by using a Canon EOS-1D digital SLR camera (Canon, Tokyo, Japan). After washing with seawater, the specimens were initially fixed and preserved in 95% ethanol. The processed specimens are temporarily stored at the Laboratory of Marine Bivalve Classification and Systematic Evolution, Institute of Oceanology, Chinese Academy of Sciences (Qingdao, Shandong, China), for subsequent preliminary processing, preliminary identification, collection of morphological data, collection of molecular data, and experimental analysis. One holotype specimen and five paratype specimens of the newly discovered species, *G. huangheensis* sp. nov., are preserved at the Marine Biological Museum of the Chinese Academy of Sciences, Institute of Oceanology, Chinese Academy of Sciences (MBMCAS) (Qingdao, Shandong, China).

We used a digital caliper for external morphological measurements, precise to 0.1 mm, to obtain morphological data. According to the manufacturer’s instructions, the TIANamp Marine Animal DNA Extraction Kit (DP324-03, Tiangen Biology, Beijing, China) was used to extract genomic DNA from the anterior and posterior adductor muscle tissues of *Glauconome.* The polymerase chain reaction (PCR) was performed on a Biorad thermal cycler to amplify a fragment of the mitochondrial cytochrome oxidase I (*COI*) and *16S rRNA* (*16S*) gene. PCR Reaction system: 25 μL of reaction system was used, with 12.5 μL of 2 × Hieff Canace ^®^ GoldPCR Master Mix (with Dye) (10102ES60, Yisheng Biotechnology, Shanghai, China), 9.5 μL ddH2O, 1 μL of forward and reverse primers, and 1 μL of DNA template [Table 1]. PCR reaction parameters: predenaturation at 95 °C for 5 min, heating denaturation at 95 °C for 40 s, annealing at 46–50 °C for 50 s, extending at 72 °C for 50 s, cycling for 35 times, extending at 72 °C for 10 min, and temporary storing at 4 °C. Primers and amplification conditions are as follows in Table 1 [2,3,12,13].

The amplicons were subjected to agarose gel electrophoresis at 1.0% for concentration detection. Then, they were submitted to Beijing Tsingke Biotech (Beijing, China), for sequencing using the Sanger method. Following trimming and manual correction, the molecular sequences were confirmed. Forty-six samples of *Glauconome* were collected, and 23 samples with distinct morphological differences were selected for bidirectional sequencing. After assembly, 23 *16S rRNA* sequences and 23 *COI* sequences were yielded. After comparative identification, 13 samples were identified as *G. angulata*, and 10 samples were identified as a new species of *G. huangheensis* sp. nov.

The genetic distances between species of the family Glauconomidae were calculated using the Kimura 2-parameter (K2P) model in MEGA 11.01, based on the *16S* and *COI* sequences [14]. The phylogenetic relationships within the family Glauconomidae were inferred using *16S rRNA* and *COI* gene sequences from five species downloaded from GenBank (with identification errors) and five self-collected species. Multiple sequence alignments of the nucleotide sequences were performed using MAFFT [15], with the *16S rRNA* aligned in normal mode, and the *COI* aligned in codon mode, using the invertebrate genetic code. Gblocks [16] was used to extract conserved regions from the alignments. The aligned sequences were concatenated into a dual-gene matrix using PhyloSuite 1.2.2 [17], and 466 tree-building analysis models were compared using ModelFinder to select the best-fit model according to the Bayesian Information Criterion (BIC). The maximum likelihood analysis was performed using IQ-TREE [18] with the best-fit model, and branch support was assessed using ultrafast bootstrap (UFB) with 1000 replicates. The Bayesian analysis was carried out in MrBayes v.3.2.6 [19] All the above analyses were completed using software platform PhyloSuite 1.2.2. The phylogenetic tree and node labels were graphically edited using iTOL [20].

## 3. Results

### 3.1. Systematics

**Order:** Venerida J. E. Gray, 1854;

**Superfamily:** Cyrenoidea J. E. Gray, 1840;

**Family:** Glauconomidae J. E. Gray, 1853;

**Genus:** *Glauconome* J. E. Gray, 1828;

**Type species:** *G. huangheensis* sp. nov.

### 3.2. Material Examined

Holotype I: Complete, Huanghekou Nature Reserve, Dongying City, Shandong Province, China, located at 95 flood plain in the low tide area of the Yellow River estuary, 29 May 2023, MBM287895.

Paratype I: Complete, Huanghekou Nature Reserve, Dongying City, Shandong Province, China, located at 95 flood plain in the low tide area of the Yellow River estuary, 29 May 2023, MBM287895.

Paratype II: Complete, Huanghekou Nature Reserve, Dongying City, Shandong Province, China, located at 95 flood plain in the low tide area of the Yellow River estuary, 29 May 2023, MBM287895.

Paratype III: Complete, Huanghekou Nature Reserve, Dongying City, Shandong Province, China, located at 96 flood plain in the low tide area of the Yellow River estuary, 29 May 2023, MBM287895.

Paratype IV: Complete, Huanghekou Nature Reserve, Dongying City, Shandong Province, China, located at 96 flood plain in the low tide area of the Yellow River estuary, 29 May 2023, MBM287895.

Paratype V: Complete, Huanghekou Nature Reserve, Dongying City, Shandong Province, China, located at 96 flood plain in the low tide area of the Yellow River estuary, 29 May 2023, MBM287895.

### 3.3. Description

**Figure 2:**
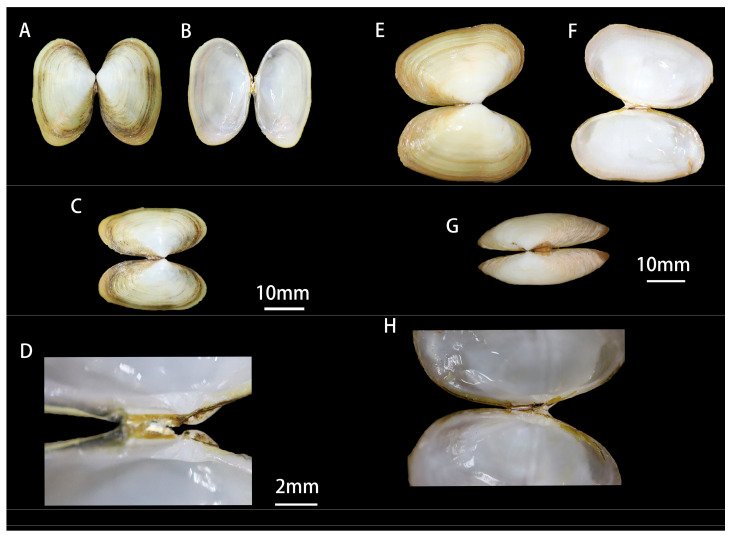
Comparison of morphological features between *G. huangheensis* sp. nov. and *G. angulata* (inner shell, outer shell, and hinge area). The left of Figure 2 (**A**–**D**) is *G. huangheensis* sp. nov., and the right (**E**–**H**) is *G. angulata*.

**Shell:** The shell of *G. huangheensis* sp. nov. is elongated and elliptical, almost rectangular, with both sides equal in length. The anterior margin is rounded and blunt, while the posterior margin is almost trapezoidal. The shell is relatively thin and slightly translucent. The apex is low and flat, located anterior to the dorsal center. The external ligament is small and thin, located posterior to the apex, with a corresponding rough area on the inner side of the shell. The umbo is slightly longer than the lunule, but the difference is not significant. Similar to the *G. angulata*, there is a distinct keel line on the posterior end of the shell. The shell color is mostly white or light green, with rough concentric growth lines on the ventral margin. There is a deep green and distinct concentric line on the ventral side of the shell. The juvenile shell is light brown, while the adult shell is darker. The inner surface of the shell is white, with a narrow and elongated hinge area. Each shell has three cardinal teeth, with the left shell having two teeth on the anterior side and the right shell having two teeth on the posterior side, with the tips of the cardinal teeth bifurcated. There are no lateral teeth [Figure 2].

**Adductor muscle scar and pallial scars:** The posterior opening is connected to a slender and flexible siphon, while the anterior adductor scar is irregularly elongated in shape and relatively small. The posterior adductor scar is slightly flattened and elliptical, with a nearly square appearance and larger size. The mantle cavity is relatively deep and elongated, with a pointed anterior end that can penetrate into the middle part of the shell and point towards the dorsal side. The pallial line gradually merges with the inner surface of the shell along the ventral margin, extending to about three-fifths of the total body length.

**Etymology:** Named after the type locality, the Yellow River estuary in Yellow River Delta Nature Reserve, Dongying City, Shandong Province, China,

**Distribution:** Currently, *G. huangheensis* sp. nov. is known only from the type locality in Yellow River Delta Nature Reserve, Dongying City, Shandong Province, China. It lives in the intertidal zone with freshwater inflow and sandy or muddy sediments.

### 3.4. Morphological Comparison

The shell of Glauconomidae is blunt and round at the anterior end and pointed and slender at the posterior end. The ventral margin is relatively straight, and the surface is covered with brownish-green, green, or white shell skin, which often peels off at the apex of the shell. The concentric growth lines are distinct, and the ventral side is folded. The hinge has three main teeth and no lateral teeth. Compared with other species in Glauconomidae, the *G. huangheensis* sp. nov. exhibits significant differences in shell color, shell sculpture, shell shape, ligament, and shell thickness.

The shell of *G. huangheensis* sp. nov. is similar to its closely related sister species *G. angulata*, but there are noticeable differences in shell color, shell surface markings, ligament size, and shell thickness. The posterior side of *G. angulata* is blunter and presents a blunt trapezoidal shape. The shell is semi-transparent, thinner, and fragile, with colors ranging from green, yellow, and light green to dark green. The ventral side exhibits dense concentric green lines, and there is a more prominent angular line on the posterior side. The ligament inside the shell is not apparent [Figure 2].

### 3.5. Molecular Support

The *16S* genetic divergence between *G. huangheensis* sp. nov. and other Glauconomidae species analyzed ranged from 2.33% (*G. angulata*) to 26.46% (*G. rugosa*) [Table 2]. The interspecies genetic distances are all greater than 0.02, supporting species independent validity.

The *COI* genetic divergence between *G. huangheensis* sp. nov. and other Glauconomidae species analyzed ranged from 2.49% (*G. angulata*) to 20.44% (*G. chinensis*) [Table 3]. The interspecies genetic distances are all greater than 0.02, supporting species independent validity.

*16S rRNA* gene sequence alignment. The comparative analysis of the *16S* sequences between *G. huangheensis* sp. nov. and *G. angulata* reveals significant differences. The sequence length is 453 base pairs, and these substitutions account for a difference of 11 nucleotides. The analysis of the *16S rRNA* gene sequences supports the classification of *G. huangheensis* sp. nov. as an independent species, with a sister species relationship to *G. angulata*.

*COI* base sequence and amino acid sequence alignment. Compared with *G. angulata*, the *COI* sequence of *G. huangheensis* sp. nov. has a difference of 16 base pairs. In the amino acid alignment, the protein-coding gene uses the Invertebrate Mitochondrial Genetic Code. Amino acid substitution analysis shows that the translated peptide chains all contain 218 amino acids. In the six *COI* r sequence (such as 23111201) of *G. huangheensis* sp. nov., it has a difference of three amino acids compared with *G. angulata*. The analysis of *COI* sequence bases and amino acids also supports *G. huangheensis* sp. nov. as an independent species and its sister relationship with *G. angulata*.

**Figure 3:**
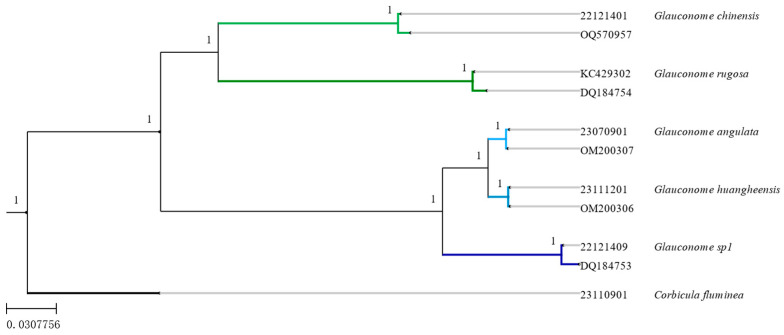
Phylogenetic relationships of Glauconomidae by the ML analysis of mitochondrial (*COI* + *16S rRNA*) sequences. The black bootstrap values ≥ 95% for the node.

**Figure 4:**
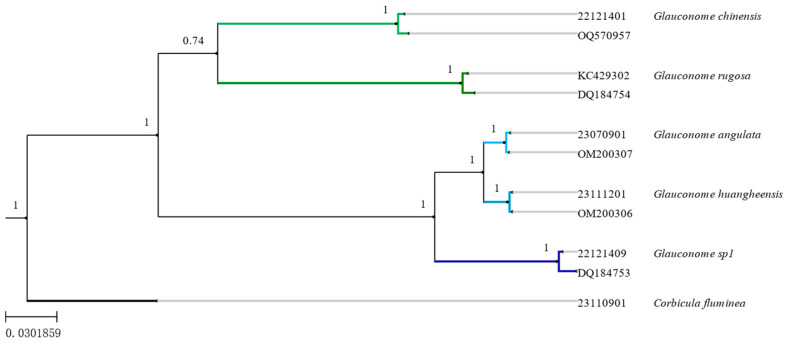
Phylogenetic relationships of Glauconomidae by the BI analysis of mitochondrial (*COI* + *16S rRNA*) sequences. The black bootstrap values ≥ 95% for the node.

Phylogenetic tree and analysis results [21]. The phylogenetic tree of the family Glauconomidae, reconstructed using maximum likelihood and Bayesian analysis based on mitochondrial *COI* and *16S rRNA* sequence data, is shown in Figure 3 and Figure 4. *Glauconome* (including *G. huanghesis* sp. nov.) formed monophyletic clades with strong support values (≥95%) [22]. *G. huangheensis* sp. nov. and *G. angulata* are closely related sister species. The results obtained by ML tree and BI tree were consistent, and the clades of the family Glauconomidae were clear and defined, which supported the effectiveness of the new species [Figure 3 and Figure 4]. Under the support of morphological and molecular phylogenetic analyses, a new species, *G. huanghesis* sp. nov., has been classified into the genus *Glauconome* of the family Glauconomidae.

## Figures and Tables

**Table 1:** Primer names, sequence information, and amplification conditions.

Primer Name	Primer Sequence	PCR Program
16SF	5′-GCCTGTTTATCAAAAACAT-3′	95 °C 5 min, 35 × (95 °C 40 s, 50 °C 50 s, 72 °C 50 s), 72 °C 10 min, 4 °C hold
16SR	5′-CCGGTCTGAACTCAGATCACG-3′	
LCO1490	5′-GGTCAACAAATCATAAAGATATTGG-3′	95 °C 5 min, 35 × (95 °C 40 s, 46 °C 50 s, 72 °C 50 s), 72 °C 10 min, 4 °C hold
HCO2198	5′-TAAACTTCAGGGTGACCAAAAAATCA-3′	

**Table 2:** Genetic distance analysis of Glauconomidae based on *16S rRNA*.

Species	G.C	G.A	G.H	G.sp1	G.R
*Glauconome chinensis*	-				
*Glauconome angulata*	0.1811	-			
*Glauconome huangheensis*	0.1931	0.0233	-		
*Glauconome*.sp1	0.1959	0.0478	0.0527	-	
*Glauconome rugosa*	0.1989	0.2449	0.2646	0.2479	-

**Table 3:** Genetic distance analysis of Glauconomidae based on *COI*.

Species	G.C	G.A	G.H	G.sp1	G.R
*Glauconome chinensis*	-				
*Glauconome angulata*	0.2102	-			
*Glauconome huangheensis*	0.2044	0.0249	-		
*Glauconome*.sp1	0.1601	0.2050	0.1968	-	
*Glauconome rugosa*	0.2262	0.1273	0.1275	0.2151	-

## Data Availability

We have uploaded sequences to GenBank (PQ660726-PQ660735, PQ663780-PQ663789), but due to temporary confidentiality of the data, it will be delayed until 20 June 2026. All the data that support the findings of this study are available in the main text or Supplementary Information.

## Supplementary Materials

The following supporting information can be downloaded at https://www.mdpi.com/article/10.3390/genes15121563/s1. Table S1: Glauconomidae GenBank Number; Table S2: Phylogenetic ML and BI tree partitions and evolutionary models selected of mitochondrial genome of Glauconomidae species.

## References

[1] (2024). World Register of Marine Species (WoRMs). *Glauconome* J. E. Gray, 1828. https://www.marinespecies.org/aphia.php?p=taxdetails&id=492515.

[2] Mikkelsen P.M., Bieler R., Kappner I., Rawlings T.A. (2006). Phylogeny of Veneroidea (Mollusca: Bivalvia) based on morphology and Molecules. Zool. J. Linn. Soc..

[3] Bieler R., Mikkelsen P.M., Collins T.M., Glover E.A., González V.L., Graf D.L., Harper E.M., Healy J., Kawauchi G.Y., Sharma P.P. (2014). Investigating the Bivalve tree of life—An exemplar-based approach combining molecular and novel morphological characters. Invertebr. Syst..

[4] Sharma P.P., Zardus J.D., Boyle E.E., González V.L., Jennings R.M., McIntyre E., Wheeler W.C., Etter R.J., Giribet G. (2013). Into the deep: A phylogenetic approach to the bivalve subclass Protobranchia. Mol. Phylogenetics Evol..

[5] Huber M. (2010). Compendium of Bivalves: A Full-Color Guide to 3300 of the World’s Marine Bivalves.

[6] Huber M. (2015). Compendium of Bivalves 2: A Full-Color Guide to the Remaining Seven Families.

[7] Liu R. (2008). Catalogue of Marine Organisms in China.

[8] Xu F., Zhang S., Wang S. (2008). An Illustrated Bivalvia Mollusca Fauna of China Seas.

[9] Zhang S. (2008). Illustrated Guide to Chinese Marine Mollusks.

[10] Wang H. (2014). Species Investigation and Research in Haizhou Bay and Laizhou Bay.

[11] Zhang H. (2018). Atlas of Common Marine Organisms in the Yellow River Delta Region.

[12] Liu Y. (2022). Systematic Evolutionary Study of the Broad Sense Veneridae.

[13] Liu Y., Ma P., Zhang Z., Li C., Chen Y., Wang Y., Wang H. (2022). The new phylogenetic relationships in Veneridae (Bivalvia: Venerida). Zool. J. Linn. Soc..

[14] Tamura K., Stecher G., Kumar S. (2021). MEGA11: Molecular Evolutionary Genetics Analysis version 11. Mol. Biol. Evol..

[15] Katoh K., Rozewicki J., Yamada K.D. (2019). MAFFT online service: Multiple sequence alignment, interactive sequence choice and visualization. Brief. Bioinform..

[16] Talavera G., Castresana J. (2007). Improvement of phylogenies after removing divergent and ambiguously aligned blocks from protein sequence alignments. Syst. Biol..

[17] Zhang D., Gao F., Jakovlić I., Zou H., Zhang J., Li W.X., Wang G.T. (2020). PhyloSuite: An integrated and scalable desktop platform for streamlined molecular sequence data management and evolutionary phylogenetics studies. Mol. Ecol. Resour..

[18] Nguyen L.T., Schmidt H.A., von Haeseler A., Minh B.Q. (2015). IQ-TREE: A fast and effective stochastic algorithm for estimating maximum-likelihood phylogenies. Mol. Biol. Evol..

[19] Ronquist F., Teslenko M., Van Der Mark P., Ayres D.L., Darling A., Höhna S., Larget B., Liu L., Suchard M.A., Huelsenbeck J.P. (2012). MrBayes 3.2: Efficient Bayesian Phylogenetic Inference and Model Choice Across a Large Model Space. Syst. Biol..

[20] Letunic I., Bork P. (2007). Interactive tree of life (iTOL): An online tool for phylogenetic tree display and annotation. Bioinformatics.

[21] Yang M., Li B., Gan Z., Dong D., Li X. (2024). A new chemosymbiotic bivalve species of the genus Acharax Dall, 1908 (Bivalvia, Solemyida, Solemyidae) from the Haima cold seep of the South China Sea. ZooKeys.

[22] Wu R., Liu L., Liu X., Ye Y., Wu X., Xie Z., Liu Z., Li Z. (2023). Towards a systematic revision of the superfamily Cyrenoidea (Bivalvia: Imparidentia): Species delimitation, multi-locus phylogeny and mitochondrial phylogenomics. Invertebr. Syst..

